# Genetic diversity and phylogenetic relationship of Angus herds in Hungary and analyses of their production traits

**DOI:** 10.5713/ab.23.0157

**Published:** 2023-08-28

**Authors:** Judit Márton, Ferenc Szabó, Attila Zsolnai, István Anton

**Affiliations:** 1 Hungarian Hereford, Angus, Galloway Association, Dénesmajor 2, H-7400 Kaposvár, Hungary; 2 Széchenyi István University, Albert Kázmér Faculty of Mosonmagyaróvár, Vár tér 2., H-9200 Mosonmagyaróvár, Hungary; 3 Hungarian University of Agriculture and Life Sciences, Institute of Animal Husbandry Sciences, Guba Sándor utca 40., H-7400 Kaposvár, Hungary

**Keywords:** Angus Cattle, Genetic Diversity, Microsatellites, Production Traits

## Abstract

**Objective:**

This study aims to investigate the genetic structure and characteristics of the Angus cattle population in Hungary. The survey was performed with the assistance of the Hungarian Hereford, Angus, Galloway Association (HHAGA).

**Methods:**

Genetic parameters of 1,369 animals from 16 Angus herds were analyzed using the genotyping results of 12 microsatellite markers with the aid of PowerMarker, Genalex, GDA-NT2021, and STRUCTURE software. Genotyping of DNA was performed using an automated genetic analyzer. Based on pairwise identity by state values of animals, the Python networkx 2.3 library was used for network analysis of the breed and to identify the central animals.

**Results:**

The observed numbers of alleles on the 12 loci under investigation ranged from 11 to 18. The average effective number of alleles was 3.201. The overall expected heterozygosity was 0.659 and the observed heterozygosity was 0.710. Four groups were detected among the 16 Angus herds. The breeders’ information validated the grouping results and facilitated the comparison of birth weight, age at first calving, number of calves born and productive lifespan data between the four groups, revealing significant differences. We identified the central animals/herd of the Angus population in Hungary. The match of our group descriptions with the phenotypic data provided by the breeders further underscores the value of cooperation between breeders and researchers.

**Conclusion:**

The observation that significant differences in the measured traits occurred among the identified groups paves the way to further enhancement of breeding efficiency. Our findings have the potential to aid the development of new breeding strategies and help breeders keep the Angus populations in Hungary under genetic supervision. Based on our results the efficient use of an upcoming genomic selection can, in some cases, significantly improve birth weight, age at first calving, number of calves born and the productive lifespan of animals.

## INTRODUCTION

The Aberdeen Angus is one of the oldest – and probably the most iconic – beef cattle breeds in the world. It derives from the Scottish counties of Aberdeenshire and Angus in northeast Scotland, where the black hornless cattle were raised by local farmers.

The first printed reference to polled cattle in Angus was made by Rev. James Playfair in 1797 in the Old Statistical Account of the parish of Bendochy [1]. Hugh Watson, William McCombie and Sir George MacPherson Grant are considered leading promoters and improvers of the Aberdeen Angus breed. Starting of the Herd Book (1862) was an important event in the history of the breed, which was followed by the institution of the Polled Cattle Society in 1879 [1].

In the 19th century, the breed rapidly spread across the whole of the United Kingdom, France and other countries, such as Argentina, Australia, Uruguay, and the United States of America. It is currently one of the most popular breeds worldwide due to the superior quality of its meat. Today, the breed is available and raised in many countries throughout the world. The number of registered Aberdeen Angus animals continues to rise year-on-year. Based on the British Cattle Movement Service (BCMS) registration results, in 2021 Aberdeen Angus became Britain’s most popular cattle breed [2]. Aberdeen Angus cattle are regarded as medium-sized animals and produce a high carcass yield of excellent quality marbled meat. The native colour is black, but more recently red colours have also emerged. Black and Red Angus are closely related breeds. The low genetic distance between them indicates a relatively recent divergence between these breeds [3]. The UK registers both Red Angus and Black Angus in the same herd book, but in some countries (e.g. the United States and Australia) they are regarded as two separate breeds.

The melanocyte-stimulating hormone receptor is of major importance in the determination of bovine coat colour. A polymorphism in the dominant *E^D^* allele of the gene is responsible for the black colour, while a frameshift mutation in homozygous *e/e* animals results in a red coat colour [4].

Black-hided Angus calves had higher average daily weight gain, required shorter fattening time to reach slaughter weight and had fewer health problems and deaths than non-black-hided calves [5]. Comparing the feeding behaviour of Black and Red Angus cattle, Wolfger et al [6] observed elevated feed intake in the case of black animals, which resulted in higher average daily weight gains. Previously, McLean and Schmutz [7] reported a faster rate of gain and better carcass quality in black cattle, which was associated with a particular melanocortin 1 receptor genotype.

Lozada-Soto et al [8] examined the consequences of genomic selection on the genetic diversity of American Angus cattle. They found significant depressive effects of inbreeding on economically important growth traits.

Results of Karamfilov [9] suggested that Aberdeen Angus cows are more docile after the age of four years. These animals express higher resistance to diseases, have stable immunity and lower treatment expenses [10].

The Angus breed was first introduced in Hungary in the 1950s to develop cross-breeding programmes among different beef cattle breeds [11]. Later, both Black and Red Angus animals were imported to Hungary on several occasions.

The Hungarian Hereford, Angus, Galloway Association (HHAGA) was founded in 1988 and since then has continued to work to improve breeding activity and preserve the superior genetic characteristics of the breed. The HHAGA has been a member of the World Hereford Council since 1990 and a member of the European Angus Forum since 2002.

Comparing reproductive performance of nine beef cattle breeds (Hungarian Sim-mental, Hereford, Aberdeen Angus, Red Angus, Lincoln Red, Limousin, Charolais, Blonde d’Aquitaine, and Shaver) Bene et al [12] found that Red Angus cows had the highest 205-day weaning weight per cow and per 100 kg cow weight (143.9 kg and 23.9 kg/100 kg, respectively). Marker assisted selection (MAS) has been used by Hungarian researchers as molecular tool in cattle breeding since 1996 [13]. In the 2000s several studies were conducted in Angus bulls in Hungary to investigate the effect of diacylglycerol acyltransferase 1, thyroglobulin, and leptin loci on the marbling of meat. Significant differences (p<0.05) were observed between genotypes in all cases, concerning fat percentage values in the longissimus dorsi and semitendinosus muscles [14,15].

Microsatellite markers are widely used in population genetics, conservation genetics, and parentage identification [16–19]. In recent years, several genetic analyses have been performed in cattle populations based on microsatellite markers [11,17,20,21]. Since no previous studies have been made concerning the genetic structure of Angus cattle in Hungary, we aimed to provide breeders with sufficient information to preserve and protect the genetic diversity of the breed and to indicate those herds, which demand special consideration by the HHAGA.

## MATERIALS AND METHODS

**Figure 1:**
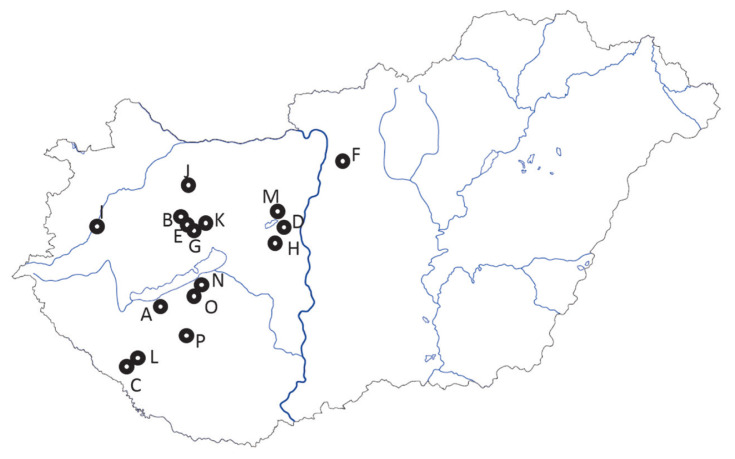
Sampling locations of 16 Angus herds in Hungary. Nearest settlement names and the coordinates are given in the Supplementary Table 1. Grey lines within the border represent the main body of rivers. Dark grey line is the Danube. The closed area, north from farms A, O, and N, south from farm G, is the lake Balaton.

A total of 1,369 Angus cows from 16 different Hungarian herds (Figure 1; Table 1; Supplementary Table S1) were investigated by genotyping 12 microsatellite markers (BM1824, BM2113, ETH3, ETH10, ETH225, INRA023, TGLA122, TGLA126, BM1818, MGTG4B, CSSM66, and CSRM60) using an automated ABI 3500 Genetic Analyzer (Applied Biosystems, Foster City, CA, USA). All of the above-mentioned microsatellite markers are recommended by the International Society of Animal Genetics (ISAG) for parentage control examinations [22]. The collection of blood samples was an integral part of the regularly executed routine parentage testing performed by trained veterinarians. The genomic DNA extraction, polymerase chain reaction and fragment length determination were completed according to the method used by Szűcs et al [17].

The number of animals, regarding the analyses of productivity data were 4,082 cows. They were arranged [23–25] into four sets according to the microsatellite based identification of A-C-M, F-J-L-N-O-P, B-D-E-G-H-I, and K groups (see RESULTS AND DISCUSSION).

Effective number of alleles, observed and expected heterozygosity values, inbreeding coefficients and principal coordinate analysis were calculated by GenAlEx [26]. Neighbour-joining tree was constructed by MEGA [27].

Identity-by-state pairwise value (IBS) between any two individuals was calculated as: ([number of markers sharing two alleles + 0.5× number of markers sharing one allele]/number of markers). Betweenness centrality, was calculated and visualised by Python 3.6 software using the libraries networkx 2.3 and matplotlib 3.1.1. Betweenness centrality of a given $\text{animal}/\text{node}=\Sigma_{s\neq v\neq t}^{v}\frac{\sigma_{st|\text{v}}}{\sigma_{st}}$ where v is the number of nodes, σ_st_ is the total number of shortest paths from node s to node t and σ_st|v_ is the number of those paths that pass through the node v.

For better visualisation of the genetic network based on IBS values and from the point of view of betweenness centrality, we reduced the number of visible edges/connections/IBS values and nodes/animals.

## RESULTS AND DISCUSSION

The observed numbers of alleles on the 12 loci under investigation ranged from 11 to 18. The average effective number of alleles was 3.201. The overall expected heterozygosity was 0.659 and the observed heterozygosity was 0.710 (Table 1). In British Angus herds, the observed heterosygosity using twelve microsatellites (n = 33) was 0.428 [28], while 50 animals with 30 microsatellites [29] yielded 0.610 value. Eleven microsatellites and 30 Angus individuals reared in Russia produced Ho with 0.665 [30]. Twenty two microsatellites on 164 Canadian Angus [31] resulted 0.630 Ho value. Old and New Type Colombia Angus (n = 29) Ho were equal to 0.734 and 0.707, respectively [32]. Ten microsatellites on 61 Columbian Angus [33] have given Ho = 0.600. The hetorosygosity on the farms studied, ranged from 0.600 (herd M) to 0.809 (herd B) which are similar to the reported values above. Only farms E, I, J, K, O, and P exceeded the reported Ho value of Colombian values.

All herds, excluding D, had more heterozygous animals than expected. Six herds (B-J-M-N-O-P) had an inbreeding coefficient lower than or equal to −0.1, and herd B had a value lower than −0.2. At the time of testing the herds, B had the highest heterozygosity value, while a small extent of heterozygote deficiency was observed only in herd D.

**Figure 2:**
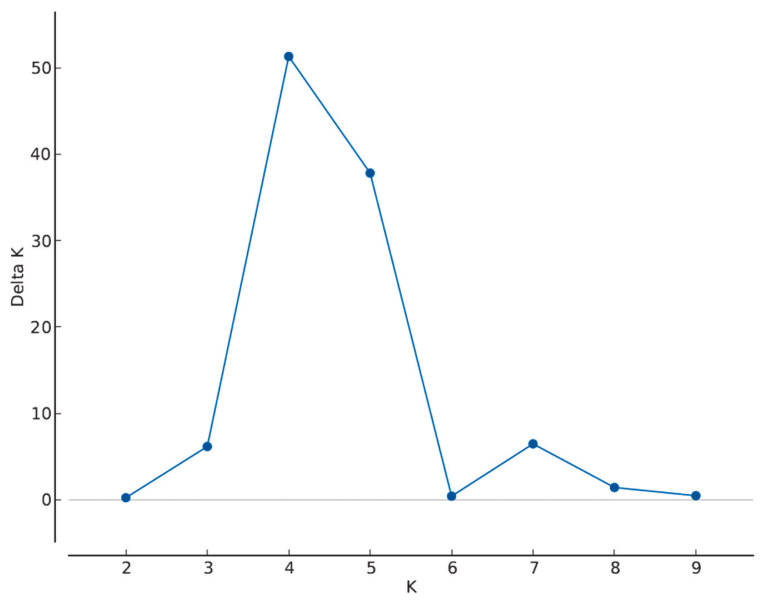
Determination of the most probable cluster number (K) of 16 Angus herds using ΔK approach on Structure lnP(D) values. The highest ΔK value is at K = 4.

**Figure 3:**
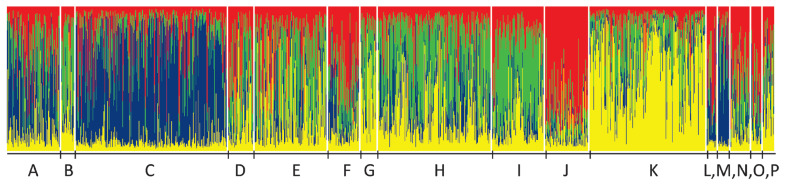
Structure plot of the herds from A to P at K= 4. Animals are represented by vertical lines, and their ratios from the identified groups are represented by different colours. Herds where the blue colour is dominant, are A-C-M. The high portion of red is given by populations F-J-L-N-O-P, and prevailing green defines B-D-E-G-H-I. Yellow overrepresentation is found in herd K.

The most probable cluster number was four (Figure 2). At K= 4 the herds were grouped as A-C-M marked mostly by blue, F-J-L-N-O-P by red, B-D-E-G-H-I by green, and K by yellow (Figure 3).

**Figure 4:**
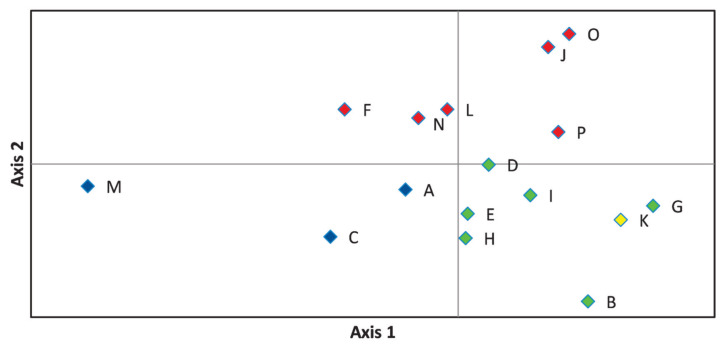
Representation of principal component analysis of estimated pairwise genetic distance values obtained by Genalex software, where axes 1 and 2 describe 33.68% and 17.88% of the total variance, respectively. Herd groups are marked by their dominant colour as given in Figure 3. Blue colour is for A-C-M. Red is given to F-J-L-N-O-P, green defines B-D-E-G-H-I, yellow denotes herd K.

On the principal coordinate plots (Figure 4) where the first and second axes account for 33.68% and 17.88% of the variance, respectively, the A-C-M and F-J-L-N-O-P herds were grouped as observed in Figure 3. The first axis differentiate between the blue and green groups, while the second divides red from blue, and red from green marked farms.

**Figure 5:**
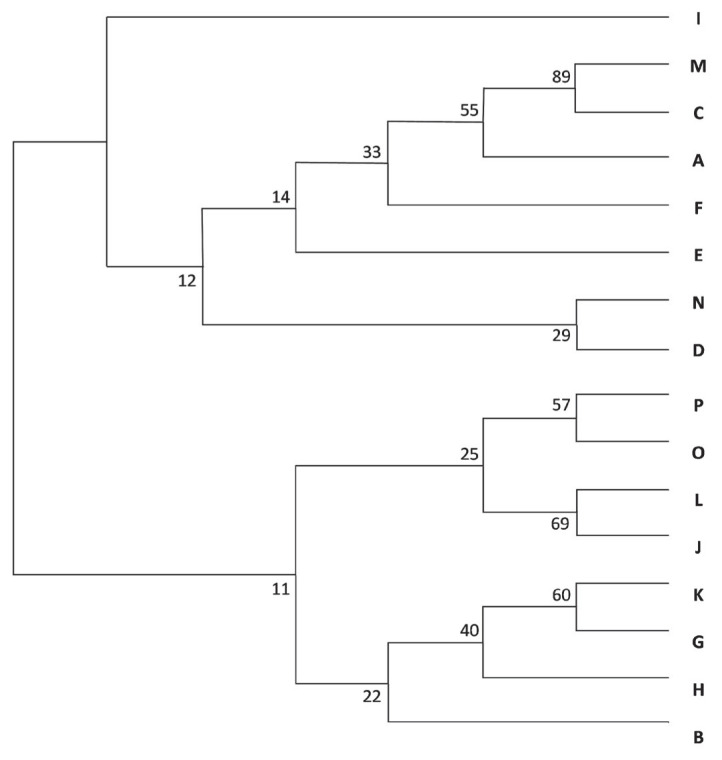
Neighbour-joining tree of herds from A to P. Numbers indicate the bootstrap values. The A, C, and M herds are on the same branch, with bootstrap values higher than 50. Among the STRUCTURE identified F-J-L-N-O-P group J, L, O, and P herds were also grouped together.

The dendrogram (Figure 5)—based on Nei’s genetic distance—placed the A-C-M group on the same branch, with bootstrap values higher than 50. From the F-J-L-N-O-P group identified via STRUCTURE and principal coordinate analyses, J-L-O-P were also grouped together.

The grouping of the herds was determined by three methods. STRUCTURE identified four groups; two of them, A-C-M and F-J-L-N-O-P, were supported by the principal coordinate analysis. The consensus tree of the population supported the existence of the A-C-M group; their bootstrap values were over 50.

**Figure 6:**
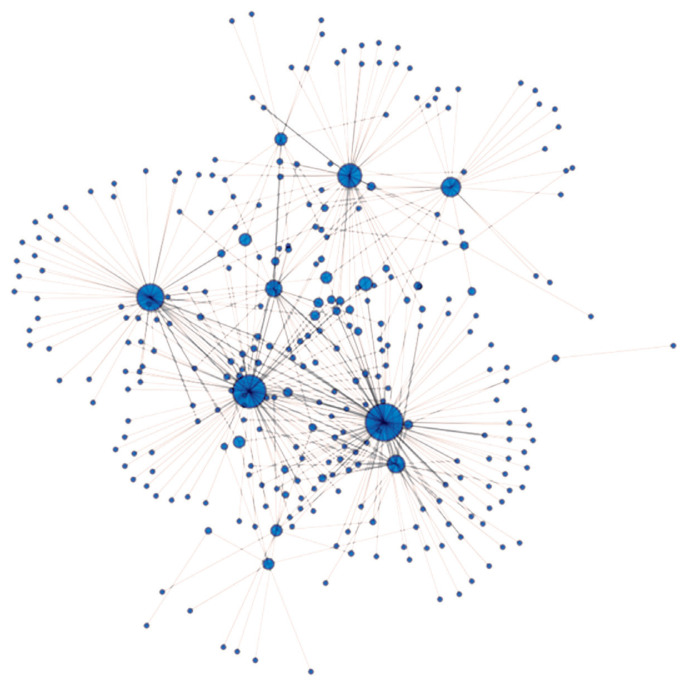
Genetic net based on identical by state values (IBS). IBS values above 0.624 are presented in the figure. Nodes/circles are the animals from herd A, and connections/edges are the pairwise IBS values between the nodes. The diameter of the nodes/animals is proportional to their betweenness centrality.

The IBS value where the network remained interconnected was 0.624. The four highest betweenness centrality values belonging to the four animals denoted by the largest blue circles on Figure 6. were 0.488, 0.375, 0.251, and 0.194. The central animals of the Angus farms under study originated from herd A. These animals have the highest betweenness centrality scores among the studied individuals, having the highest genetic similarity to other animals and to each other (Figure 6). So herd A has animals sharing their state of genetic background with most of their herd companions. By comparing the overall patterns (Figure 6) of herd A (n = 97) and the pattern obtained by the same methods on Hungarian Merino sheep (n = 138, Figure 4A [34]) it may be noted the appearance of a wheel-like structure. The reason for similar genetic net patterns of the Angus cattle and the Merino sheep [34] is that both are maintained for commercial reasons, so parameters for production traits are primordial. Such wheel-like structures might be common in industrial breeds and might not be characteristic for the breeds where maintenance of diversity is of cardinal importance. That assumption requires further tests beyond the scope of the current study.

Since the genetic analyses were performed blindly, without the knowledge of the phenotypic appearance of the animals and performance data, we examined the history of the studied herds, with the support of the HHAGA. During that inspection, we found similarities within the four groups identified by structure clustering. In A-C-M herds, Canadian Aberdeen Angus bulls were preferred for inseminations. Their individuals were mostly red-coloured variants (over 95%) with large body size. Herd M derives from herd C. Herds F-J-L-N-O-P mostly consist of British red Aberdeen Angus-type animals. Inseminating bulls were coming from herds A, C and D. Herd N’s founders are derived from herds A and P, herd O is the descendant of N and P, while P contains German Fleckvieh ancestors in their maternal line. The common feature of the studied B-D-E-G-H-I groups is that they belong to the traditional black Aberdeen Angus type. Inseminating bulls are mostly provided by herd D, which has undergone a cross with Blonde d’Aquitaine cattle. The fourth group contains one herd, K, of which 20% are of the Limousin bloodline. In summary, A-C-M is composed of red, large, Canadian-type animals; the F-J-L-N-O-P group contains the traditional red, British-type; the B-D-E-G-H-I group has traditional black, British-type animals, while K’s ancestors can be traced back to cows of English origin.

Since the genetic differences could be explained by the different types of animals, we became interested in comparing birth weight, age at first calving, number of calves born and productive lifespan between the identified groups. The Kruskal-Wallis test indicated that birth weight, age at first calving and productive lifespan were different among the groups defined by microsatellite data. The Dunnett T3 test between the pairs of groups showed significant differences at the p<0.05 level (Table 2).

Birth weight of Angus calves in Canada averaged 34 kg and was positively correlated with post-weaning daily weight gain [35]. Offspring from British (Angus and Hereford) sires were heavier (40.5 kg) than calves from Norwegian Red, Swedish Red and White and Friesian sires. Moreover, authors observed that effect of sire breed in case of birth weight was significant (p<0.001) [36]. Average birth weight of female Angus calves in Bulgaria was 31.6 kg [37]. The mean birth weight of progeny born to dairy cows which were artificially inseminated to Angus and Hereford bulls in New Zealand was 36.8 kg [38]. Birth weight values reported in present study are lower compared to those cited from literature.

In the USA age at first calving is expected to be 22 to 24 months of age in the majority of Bos taurus heifers (e.g. Angus, Hereford, Charolais). Heifers that first calved at two vs. three years of age, produced an average of 0.7 more calves in their lifetime [39], or in other respects produced 138 kg more of weaned calf weight in their lifetime [40].

When estimating genetic parameters for the age at first calving and first calving interval in the Czech beef cattle population low to moderate heritability of these traits were found. In case of Angus heifers age at first calving averaged 756.1 days [41].

Mean values regarding age at first calving of Angus cows in Hungary was 2.76 years (1,007 days), while longevity (productive life) proved to be 8.28 years (3,022 days) [42]. Later productive life of Angus cows in Hungary was estimated at 8.14 years (2,971 days) [43]. This range is slightly shorter than longevity of group K in the present study but exceeds the results of the three other groups.

Concerning the impact of cow age on lifetime productivity of female offspring it has been found that calves born to five-year-old (or older) Angus cows had increased productivity compared to those born to four-year-old (or younger) dams [44]. When investigating the effect of calving period on the lifetime reproductive performance and productivity of Angus cows in Canada it has been concluded that heifers calving early vs calving later in their first calving season had increased pregnancy rates and weaned more calves [45].

## CONCLUSION

Considering that no previous studies have been made on the genetic structure of Angus herds in Hungary, the results described here could be incorporated by HHAGA into ongoing and future breeding programmes to protect and preserve the genetic diversity of the breed. Identification of trait differences among the identified groups could pave the way of the targeted and efficient use of an upcoming genomic selection. For example, in herds A-C-M, birth weight, age at first calving, and the productive lifespan require substantial improvement.

## Figures and Tables

**Table 1:** Population codes (Pop code), number of animals (N), effective number of alleles (Ne), observed (Ho), and expected (He) heterozygosity values, and inbreeding coefficients (Fis) of the investigated herds

Pop code	N	Ne	Ho	He	Fis
A	97	3.149	0.678	0.656	−0.031
B	24	3.239	0.809	0.671	−0.210
C	278	3.276	0.660	0.657	−0.004
D	46	3.356	0.668	0.684	0.023
E	132	3.588	0.742	0.711	−0.046
F	57	3.191	0.683	0.657	−0.044
G	29	3.253	0.695	0.671	−0.030
H	207	2.981	0.685	0.642	−0.068
I	95	3.337	0.739	0.689	−0.071
J	79	3.343	0.743	0.678	−0.100
K	213	3.614	0.746	0.708	−0.051
L	18	2.949	0.690	0.627	−0.095
M	20	2.569	0.600	0.531	−0.133
N	35	3.023	0.726	0.634	−0.140
O	18	3.198	0.759	0.670	−0.135
P	21	3.152	0.738	0.656	−0.129
	1369	3.201	0.710	0.659	−0.079

**Table 2:** Distribution of mean values of birth weight (kg), age at first calving (day), number of calves born (head) and productive lifespan (day) and their ±standard errors among the four groups (A-C-M, F-J-L-N-O-P, B-D-E-G-H-I, and K) identified by Structure-clustering

Measured traits	A-C-M	F-J-L-N-O-P	B-D-E-G-H-I	K
birth weight (kg)	25.9^a^±2.7	29.3^b^±5.3	27.2^c^±4.4	27.6^abc^±5.9
age at first calving (d)	869^a^±206	829^b^±156	891^d^±226	945^abd^±320
number of calves born	4.8^a^±3.6	5.9^b^±3.5	6.3^bc^±3.6	8.4^c^±4.1
productive lifespan (d)	1,996^a^±1,535	2,221^a^±1,677	2,563^d^±1,520	3,556^c^±1,758

a–d Means with different uppercase letters in the same row are significantly different from each other at p<0.05.

## References

[1] Macdonald J, Sinclair J (1910). History of Aberdeen-Angus cattle.

[2] British Cattle Movement Service [Internet] Registrations reveal Aberdeen Angus as Britain’s most popular cattle breed.

[3] Kuehn L (2010). Relationships of beef breeds using the 50k chip.

[4] Klungland H, Vage DI, Gomez-Raya L, Adalsteinsson S, Lien S (1995). The role of melanocyte-stimulating hormone (MSH) receptor in bovine coat color determination. Mamm Genome.

[5] Laudert SB (2010). Factors that determine feedlot profit [Internet]. Beef Magazine.

[6] Wolfger B, Quinn C, Torres GW, Taylor M, Orsel K (2016). Comparison of feeding behavior between black and red Angus feeder heifers. Can J Anim Sci.

[7] McLean KL, Schmutz SM (2009). Associations of melanocortin 1 receptor genotype with growth and carcass traits in beef cattle. Can J Anim Sci.

[8] Lozada-Soto EA, Maltecca C, Lu D (2021). Trends in genetic diversity and the effect of inbreeding in American Angus cattle under genomic selection. Genet Sel Evol.

[9] Karamfilov S (2022). Study on the temperament of cows of the Aberdeen Angus cattle breed. Czech J Anim Sci.

[10] Hine BC, Bell AM, Niemeyer DDO (2019). Immune competence traits assessed during the stress of weaning are heritable and favorably genetically correlated with temperament traits in Angus cattle. J Anim Sci.

[11] Horn A, Szmodits T, Bodó L (1959). Experiments related to the performance of crosses among Angus and Simmental cattle (Kísérletek az angus és magyartarka szarvasmarha haszonállatelőállító keresztezésére I.), Anim Breeding (Állattenyésztés), Budapest. Hungary.

[12] Szabolcs B, Norma H, Miklós L, Ferenc S (2013). Reproductive performance of beef cattle with different genotypes kept under extensive conditions between 1999–2011. Hung J Anim Prod.

[13] Zsolnai A, Fésüs L (1996). Simultaneous analysis of bovine K-casein and BLAD alleles by multiplex PCR followed by parallel digestion with two restriction enzymes. Anim Genet.

[14] Anton I, Kovács K, Fésüs L (2008). Effect of DGAT1 and TG gene polymorphism on intramuscular fat and milk production traits in different cattle breeds in Hungary. Acta Vet Hung.

[15] Anton I, Kovács K, Holló G (2011). Effect of leptin, DGAT1 and TG gene polymorphisms on the intramuscular fat of Angus cattle in Hungary. Livest Sci.

[16] Amigues Y, Boitard S, Bertrand C (2011). Genetic characterization of the Blonde d’Aquitaine cattle breed using microsatellite markers and relationship with three other French cattle populations. J Anim Breed Genet.

[17] Szűcs M, Szabó F, Bán B (2019). Assessment of genetic diversity and phylogenetic relationship of Limousin herds in Hungary using microsatellite markers. Asian-Australas J Anim Sci.

[18] Bhargava A, Fuentes FF (2010). Mutational dynamics of microsatellites. Mol Biotechnol.

[19] Guichoux E, Lagache L, Wagner S (2011). Current trends in microsatellite genotyping. Mol Ecol Resour.

[20] Mahgoub O, Babiker HA, Kadim IT (2013). Disclosing the origin and diversity of Omani cattle. Anim Genet.

[21] Zsolnai A, Kovács A, Anton I (2014). Comparison of different Hungarian grey herds as based on microsatellite analysis. Anim Sci Pap Rep.

[22] ISAG species panel [Internet] Further information of interest concerning the ISAG comparison tests [cited 2023 Feb 16].

[23] Hubisz MJ, Falush D, Stephens M, Pritchard JK (2009). Inferring weak population structure with the assistance of sample group information. Mol Ecol Resour.

[24] Evanno G, Regnaut S, Goudet J (2005). Detecting the number of clusters of individuals using the software STRUCTURE: a simulation study. Mol Ecol.

[25] Earl DA, von Holdt BM (2012). STRUCTURE HARVESTER: a website and program for visualizing STRUCTURE output and implementing the Evanno method. Conserv Genet Resour.

[26] Peakall R, Smouse PE (2006). Genalex 6: genetic analysis in Excel. Population genetic software for teaching and research. Mol Ecol Notes.

[27] Tamura K, Stecher G, Kumar S (2021). MEGA11: Molecular evolutionary genetics analysis version 11. Mol Biol Evol.

[28] Machugh DE, Loftus RT, Bradley DG (1994). Microsatellite DNA variation within and among European cattle breeds. Proc R Soc Lond B Biol Sci.

[29] Wiener P, Burton D, Williams JL (2004). Breed relationships and definition in British cattle: a genetic analysis. Heredity (Edinb).

[30] Alsalh MA, Bakai A, Feyzullaev FR (2021). Comparative characteristics of the genetic structure of the Syrian cattle breed compared to Holstein and Aberdeen-Angus breeds. J Adv Vet Anim Res.

[31] Carruthers CR, Plante Y, Schmutz SM (2011). Comparison of Angus cattle populations using gene variants and microsatellites. Can J Anim Sci.

[32] Moreno-Sierra AM, Cerón-Muñoz MF, Soto-Calderón ID (2020). Population genetic structure of two herds of Aberdeen Angus cattle breed in Colombia. Rev Colomb Cienc Pecu.

[33] Montoya AE, Cerón-Muñoz MF, Moreno MA (2010). Genetic characterization of the Hartón del Valle, Angus, Brangus, Holstein, and Senepol cattle breeds in Colombia, using ten microsatellite markers. Rev Colomb Cienc Pecu.

[34] Zsolnai A, Egerszegi I, Rózsa L (2023). Position of Hungarian Merino among other Merinos, within-breed genetic similarity network and markers associated with daily weight gain. Anim Biosci.

[35] Bailey CB, Mears GJ (1990). Birth weight in calves and its relation to growth rates from birth to weaning and weaning to slaughter. Can J Anim Sci.

[36] Casas E, Thallman RM, Cundiff LV (2012). Birth and weaning traits in crossbred cattle from Hereford, Angus, Norwegian Red, Swedish Red and White, Wagyu, and Friesian sires. J Anim Sci.

[37] Nikolov V, Karamfilov S (2020). Growth of female calves of the Aberdeen Angus cattle breed reared in an organic farm. Scientific Papers. Series D. Animal Science.

[38] Coleman L, Back P, Blair H, López-Villalobos N, Hickson R (2021). Sire effects on birth weight, gestation length, and pre-weaning growth of beef-cross-dairy calves: a case study in New Zealand. Dairy.

[39] Morris CA (1980). A review of relationships between aspects of reproduction in beef heifers and their lifetime production: 1. Associations with fertility in the first joining season and with age at first joining. Anim Breed Abstr.

[40] Nunez-Dominguez R, Cundiff LV, Dickerson GE, Gregory KE, Koch RM (1991). Lifetime production of beef heifers calving first at two vs three years of age. J Anim Sci.

[41] Brzáková M, Čítek J, Svitáková A, Veselá Z, Vostrý L (2020). Genetic parameters for age at first calving and first calving interval of beef cattle. Animals.

[42] Dákay I, Márton D, Bene S, Kiss B, Zsuppán Z, Szabó F (2006). The age at first calving and the longevity of beef cows in Hungary. Arch Tierz.

[43] Szabó F, Dákay I (2009). Estimation of some productive and reproductive effects on longevity of beef cows using survival analysis. Livest Sci.

[44] Wellnitz KR, Parsons CT, Dafoe JM (2022). Impacts of dam age on lifetime productivity of angus replacement beef females. Animals.

[45] Damiran D, Larson KA, Pearce LT (2018). Effect of calving period on beef cow longevity and lifetime productivity in western Canada. Transl Anim Sci.

