# Supplementary material for: Genetic diversity and phylogenetic relationship of Angus herds in Hungary and analyses of their production traits
Source: Anim Biosci. 2023 Aug 28;37(2):184–92. doi: 10.5713/ab.23.0157 (PMC10766482; doi:10.5713/ab.23.0157)
Supplement: Supplementary file 1 [file ab-23-0157-Supplementary-Table-1.pdf]

Supplementary Table 1.
Codes of Angus herds used in the main text, settlement names and GPS positions

Herd codes	Settlement names	GPS coordinates
A	Sáripuszta	46.627720, 17.418806
B	Pápa	47.325997, 17.511661
C	Somogyudvarhely	46.164760, 17.197048
D	Adony	47.108624, 18.859555
E	Pápakovácsi	47.264738, 17.512172
F	Vácegres	47.680059, 19.365456
G	Döbrönte	47.234702, 17.546328
H	Bernátkút	46.997896, 18.847972
I	Gérce	47.204782, 17.028181
J	Románd	47.446239, 17.787094
K	Döbrönte	47.237805, 17.541885
L	Somogyszob	46.267072, 17.302678
M	Gárdony	47.163852, 18.646291
N	Lulla	46.785724, 18.020383
O	Németsűrűpuszta	46.639200, 17.912109
P	Rákópuszta	46.348676, 17.949283
